# Aesthetic Profiling and Exploratory Composting Screening of Wood-Fiber Biocomposites Bonded with Spent Coffee Grounds and Ammonium Lignosulfonate

**DOI:** 10.3390/ma19061077

**Published:** 2026-03-11

**Authors:** Aleksandrina Kostadinova-Slaveva, Viktor Savov, Petar Antov, Boyka Malcheva, Ekaterina Todorova, Jansu Yusein, Viktoria Dudeva, Georgi Ivanov

**Affiliations:** 1 Faculty of Ecology and Landscape Architecture, University of Forestry, 1797 Sofia, Bulgaria; etodorova@ltu.bg; 2 Center of Competence “Clean Technologies for Sustainable Environment—Water, Waste, Energy for Circular Economy”, 1a James Bourchier Blvd., 1164 Sofia, Bulgaria; 3 Faculty of Forest Industry, University of Forestry, 1797 Sofia, Bulgaria; p.antov@ltu.bg (P.A.); yusein@ltu.bg (J.Y.); v.dudeva@ltu.bg (V.D.); georgi_ivanov@ltu.bg (G.I.); 4 Faculty of Forestry, University of Forestry, 1797 Sofia, Bulgaria; b_maltcheva@ltu.bg

**Keywords:** sustainable biocomposites, wood fibers, spent coffee grounds, ammonium lignosulfonate, bio-based binder, CIE L*a*b* (CIELAB), color difference (ΔE*), composting, bio-degradation, circular economy

## Abstract

Spent coffee grounds (SCGs) and lignin-derived binders, such as ammonium lignosulfonate (ALS), are increasingly being explored as renewable resources to reduce reliance on conventional formaldehyde-based resins in wood-fiber biocomposites. Although prior work has shown that SCG–ALS adhesive systems can achieve promising mechanical performance, two practical aspects essential for industrial applications and circular design remain insufficiently explored: a predictable and reproducible visual appearance and credible end-of-life options. In this study, sustainable wood-fiber biocomposites bonded with SCG and ALS were assessed from an aesthetic performance and end-of-life perspective. Color was quantified in the CIE L*a*b* (CIELAB) space and expressed as total color difference (ΔE*) relative to a reference panel. Increasing total SCG + ALS content from 40 to 75 wt.% based on oven-dry fibers produced pronounced darkening, with lightness decreasing from L* = 47.1 to 34.3 and ΔE* increasing from 18.38 to 32.51. Short-term composting behavior was explored by embedding fragments from formulations with 40–60 wt.% total SCG + ALS (based on oven-dry fibers; equal SCG/ALS shares) into a mixed organic substrate adjusted to an initial C/N ≈ 30 and monitored for 30 days in pots and trays. The process remained predominantly mesophilic (≈14–22 °C); nevertheless, visible microbial colonization and progressive surface degradation were observed, indicating susceptibility to biological activity under moist, nutrient-rich conditions. Overall, the results show that SCG–ALS content strongly governs the visual identity of the biocomposites and suggest composting-oriented routes as a potential end-of-life direction at an exploratory level, while highlighting the need for standardized compostability assessment and longer-term monitoring to substantiate circularity claims.

## 1. Introduction

The transition toward a circular bioeconomy has intensified research on wood-fiber biocomposites that valorize industrial and agri-food residues and reduce dependence on fossil-derived, formaldehyde-based adhesives. Quantitatively, the relevance of wood-based composites is underlined by the economic and industrial scale of the forest-based sector. FAO estimates that the formal forest sector contributes more than USD 1.5 trillion to national economies globally [1]. In terms of material throughput, global production of wood-based panels alone reached 367 million m^3^ in 2020 (including plywood, particleboard/OSB and fiberboard), reflecting their central role in construction, furniture, and interior applications [2]. In addition, FAO reports that worldwide trade in wood and paper products remained at the hundreds-of-billions USD scale, with exports amounting to USD 482 billion in 2023 [3]. In conventional wood-based composites, urea–formaldehyde (UF) and related aminoplastic resins remain dominant due to their low cost, rapid curing, and excellent mechanical performance; however, increasingly stringent environmental regulations and persistent health concerns linked to formaldehyde emissions continue to stimulate the development of novel, ultra-low-emitting formaldehyde urea formaldehyde (ULEF-UF) and no-added formaldehyde adhesive systems for biocomposite manufacturing [4,5,6,7]. In parallel, high-volume organic side streams, such as spent coffee grounds (SCGs), have attracted increasing interest within resource-efficiency frameworks, as landfilling can lead to anaerobic decomposition and greenhouse gas emissions. In contrast, valorization options can upgrade SCG into value-added products [8,9].

SCG is a heterogeneous lignocellulosic residue containing cellulose, hemicellulose, lignin, lipids, and bioactive compounds, with composition influenced by coffee variety and extraction conditions [8,9,10,11,12]. Consequently, SCG has been investigated in a broad range of applications, including adsorption media and chemically/thermally activated materials [13], hydrothermal liquefaction routes for bioenergy and biochemicals [14], and densified biofuel pellets, where carbonization can further increase heating value [15,16,17,18,19,20,21,22,23,24]. Within materials engineering, SCG is increasingly incorporated as a bio-filler in thermoplastics and biopolymers; studies in polypropylene and polylactic acid (PLA) systems show that SCG content and interface control influence dispersion, mechanical performance, and moisture sensitivity [24,25]. Importantly, the moisture sensitivity of SCG-containing systems should be interpreted in the broader context of hygroscopic lignocellulosic polymers. At the nanoscale, increasing relative humidity has been shown to reduce the Young’s modulus of model films prepared from cellulose, hemicelluloses, and lignin, illustrating how water sorption modifies mechanical response and interfacial interactions in lignocellulosic networks [26]. Consistently, recent SCG-filled biopolymer composites report that moisture uptake depends strongly on formulation variables such as SCG content and particle size, and that optimizing filler characteristics is critical to balancing stiffness–ductility trade-offs together with moisture sensitivity [27]. More generally, hydrophilic lignocellulosic fillers can promote dimensional instability/defects if processing and moisture management are not adequately controlled, while surface modification or compatibilization strategies may partially recover hydrophobicity. These observations align with the broader biocomposite literature, where combining constituents is routinely used to tune wettability, degradation behavior, and mechanical performance for targeted applications [28,29,30,31].

Recent work extends SCG utilization into additive manufacturing, where SCG-filled PLA composites are considered for functional or building-related applications, and the natural coloration of SCG is often presented as an aesthetic feature rather than a drawback [32,33,34]. Taken together, these streams of research demonstrate the versatility of SCG, but they also underline a practical challenge: the same chemical complexity that enables multiple valorization pathways can introduce variability in appearance and end-of-life behavior.

From the binder perspective, lignin-derived systems such as lignosulfonates are particularly attractive because lignin is produced in large quantities yet remains underutilized beyond low-value energy applications. Ammonium lignosulfonate (ALS) is readily water-soluble over a wide pH range and can be formulated into adhesive systems; nevertheless, its relatively low intrinsic reactivity often requires targeted formulation approaches, e.g., crosslinkers, catalysts, or hybridization with co-binders, to achieve industrially relevant curing kinetics and bond performance [5]. Accordingly, lignin-based adhesives have also been investigated as partial or full replacements for conventional resins in wood composites, with particular attention to mechanical performance and formaldehyde emission mitigation [6,7]. These developments provide a strong platform for formaldehyde-free or reduced-formaldehyde wood-fiber biocomposites, yet they also highlight that binder selection and formulation can influence not only strength and durability, but also processing behavior, penetration, density profiles, and ultimately product aesthetics and consistency.

Against this background, SCG combined with ALS represents a compelling, circularly motivated binder concept that couples an abundant consumer-derived biowaste with an industrial lignin derivative. In preceding work, sustainable wood-fiber biocomposites formulated with SCG and ALS were developed and characterized, demonstrating that suitably designed SCG-ALS binder systems can deliver promising overall property profiles and functional performance [4]. However, moving beyond proof of concept toward practical application requires stronger evidence in two main areas. First, predictable appearance and color uniformity are often decisive for market acceptance, particularly in interior and consumer-facing applications. In SCG-containing systems, however, inherent dark pigments and process-induced thermal reactions can markedly alter color and may amplify visual variability and heterogeneity across panels [35,36,37,38,39].

Second, credible circularity claims require realistic end-of-life considerations. Although SCG is frequently proposed for composting and soil-related applications, the actual outcomes are highly process-dependent, varying with the chosen route (e.g., aerated composting versus vermicomposting), substrate recipe and C/N balance, moisture and aeration control, and the degree of process maturity and stabilization [20,21]. Soil and agronomic studies further show that SCG-derived amendments can affect soil fertility and plant nutrition, but they also indicate that stabilization and proper treatment are important to mitigate phytotoxicity risks linked to undecomposed organics [17,18,19,22,23]. For SCG–ALS wood-fiber biocomposites, in particular, there is still limited evidence on how readily material fragments are colonized and degraded under composting-like conditions, and thus on their susceptibility to biological activity at end-of-life.

This leads to a clear problem statement: even when bio-based wood-fiber biocomposites demonstrate adequate mechanical performance, their adoption and circular design remain constrained by limited quantitative evidence on predictable aesthetic outcomes and plausible end-of-life behavior under biologically active conditions. In other words, the material system requires a bridge between performance-centric development and application-centric requirements such as appearance control and end-of-life feasibility.

Accordingly, the present study addresses the following research gap: for wood-fiber biocomposites bonded with an SCG–ALS system, there is a lack of systematic quantification linking formulation (SCG + ALS content) to objective color metrics, and while SCG-filled polymer composites and 3D-printable formulations report darkening trends and sometimes discuss biodegradation qualitatively [24,25,26,27,28], comparable formulation-resolved colorimetric datasets for SCG–ALS wood-fiber biocomposites remain scarce. Similarly, the SCG composting literature largely focuses on SCG as a feedstock rather than SCG incorporated into a lignin-bonded biocomposite architecture [20,21].

The novelty of this follow-up study lies in providing an integrated aesthetic and exploratory composting-oriented screening for the SCG–ALS wood-fiber biocomposite system introduced previously [1]. Specifically, formulation-dependent color is quantified using CIE L*a*b* (CIELAB) coordinates and ΔE* across an extended range of SCG + ALS contents, and short-term composting response is explored using a mixed organic substrate adjusted to an initial C/N close to common composting targets, with comparison between two simple container geometries to reflect aeration/mixing differences. By framing these results in the context of SCG composite research and the SCG composting/soil-amendment literature [8,9,17,18,19,20,21,24,25,26,27,28], the study provides practical guidance for designing sustainable wood-fiber biocomposites whose appearance and end-of-life pathways are considered alongside material performance.

To address this gap, the present work investigates SCG–ALS wood-fiber biocomposites along two application-relevant dimensions. First, formulation-dependent appearance is quantified through CIE Lab* (CIELAB) coordinates and ΔE* relative to a reference panel across a broad range of total SCG and ALS contents. Second, an exploratory end-of-life screening is performed using a laboratory composting setup in which biocomposite fragments are embedded in a mixed organic substrate adjusted to an initial C/N ratio close to common composting targets, and monitored for temperature evolution and observable degradation. The main contributions are: (i) a formulation-resolved, objective colorimetric dataset linking SCG and ALS content to visual identity and (ii) initial evidence of biological susceptibility under composting-like conditions, which defines clear targets for subsequent standardized compostability validation.

This manuscript builds upon our previously published study on the development and characterization of SCG–ALS wood-fiber biocomposites, where the manufacturing route and the main physical, mechanical, and thermal performance trends were reported in detail [4]. In the present work, we extend that baseline by addressing two application-driven aspects that were not covered previously, namely (i) formulation-resolved colorimetric characterization and aesthetic reproducibility using the CIELAB approach and (ii) end-of-life assessment through mesophilic composting-oriented screening. Therefore, while selected methodological elements are consistent with our earlier work to ensure comparability, the novelty here lies in linking composition to appearance control and to practical disposal scenarios relevant to circular material design.

## 2. Materials and Methods

### 2.1. Raw Materials

Spent coffee grounds (SCGs) were collected from local coffee shops immediately after beverage preparation and transported to the laboratory in sealed containers. Because fresh SCGs are highly susceptible to microbial growth during storage, the material was spread in thin layers and air-dried at ambient conditions, with periodic mixing to accelerate moisture removal, until a stable dry state suitable for handling and weighing was reached. The dried SCG was then stored in airtight containers to minimize moisture uptake and prevent contamination.

Commercial ammonium lignosulfonate (ALS; trade name D-947L; CAS No. 8061-53-8) was supplied by Borregaard (Sarpsborg, Norway). According to the manufacturer’s technical data, ALS had a total solids content of 48.6%, ammonium content of 4.1%, sodium content of 0.1%, total sulfur content of 6.8%, and HPLC sugars of 20%. The ALS solution exhibited a pH of 4.5, a viscosity of 400 cps at 25 °C, a specific gravity of 1.220 g·cm^−3^, and a boiling point of 104 °C. Thermomechanical wood fibers (TMFs) were obtained by the Asplund process (Defibrator L56, Valmet, Espoo, Finland) at an industrial plant (Kronospan Bulgaria EOOD, Burgas, Bulgaria), using a furnish composed of approximately 40% hardwoods (European beech and Turkish oak) and 60% softwoods (primarily Norway spruce and Scots pine).

### 2.2. Panel Manufacturing and Conditioning

Biocomposites were manufactured following the previously established manufacturing route [1], with the formulation matrix and testing sequence adapted to support an expanded aesthetic evaluation and an exploratory composting-oriented screening. Panels were manufactured in a target format of 400 mm × 400 mm × 6 mm at a nominal density of 750 kg·m^−3^. The binder fraction was defined as the combined mass of SCG and ALS, expressed relative to the oven-dry mass of the wood fibers. The main formulation series employed equal shares of SCG and ALS within the binder fraction, resulting in total SCG + ALS contents of 40%, 50%, 60%, 70%, and 75% (i.e., 20/20, 25/25, 30/30, 35/35, and 37.5/37.5 wt.% of SCG/ALS, respectively, based on oven-dry fibers). A reference panel type bonded with a conventional urea–formaldehyde resin was also produced for benchmarking of key properties.

For panel preparation, ALS was diluted to a working concentration, and SCG was introduced as an aqueous suspension so that both components could be distributed more uniformly within the fiber mass. Blending was performed in a laboratory mixer for several minutes, followed by additional stirring when needed to improve homogeneity prior to mat formation. The furnish was formed into a mat using a forming frame, gently pre-pressed to reduce bulk and improve handling, and then hot-pressed. Based on preliminary trials (including an unsuccessful attempt at very high overall binder content due to poor self-support of the mat), the upper binder level was limited to 75% to maintain manufacturability and structural integrity.

Hot pressing was carried out at 160 °C using a three-stage pressure schedule designed to balance consolidation, vapor release, and surface quality: an initial high-pressure stage at 1.8 MPa for 1 min, followed by 0.6 MPa for 10 min, and a final stage at 0.4 MPa for 4 min (total press time 15 min).

### 2.3. Physical and Mechanical Testing

After pressing, panels were cooled to room temperature and conditioned under standard laboratory conditions prior to specimen preparation and testing (conditioning to constant mass was applied before property evaluation). Specimens for physical–mechanical testing were cut from conditioned panels and assessed using standard methods consistent with the previous study [4], including density, thickness swelling, and water absorption after immersion; bending strength/modulus; and internal bond strength, where applicable. Density, thickness swelling (TS), and water absorption (WA) were determined according to the relevant EN standards (EN 323 [40], EN 317 [41], EN 310 [42], and EN 319 [43]) and as described in our previous work [4]. For clarity, TS and WA are reported as percentage changes after water immersion relative to the initial thickness/mass, respectively. Bending properties (MOR and MOE) were obtained from three-point bending tests following the respective standard definitions, and internal bond strength (IB) was calculated as the maximum tensile load at failure divided by the bonded area.

### 2.4. Colorimetric Measurements

**Figure 1:**
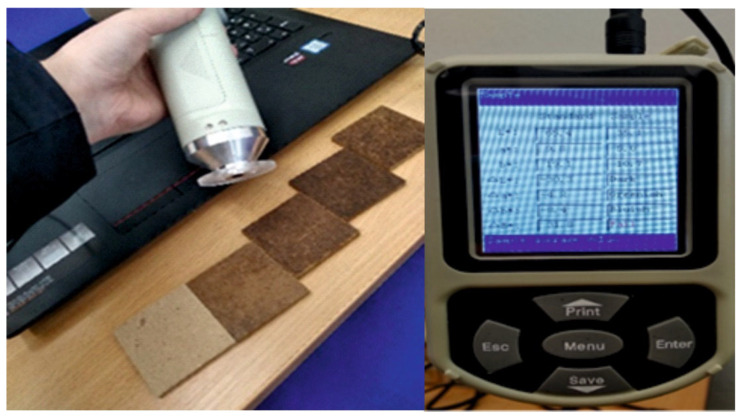
Schematic of the experimental workflow and sampling for aesthetic and exploratory composting-oriented screening.

Aesthetic characterization focused on objective color measurements using a portable colorimeter (SC-30) operating with a 45/0 optical geometry, 10° observer angle, and an 8 mm measurement aperture; the instrument reports CIE L*a*b* (CIELAB) coordinates and derived color-difference metrics (ΔE*ab) and was used to quantify surface appearance and differences among formulations, as shown in Figure 1. Color measurements were performed in triplicate (*n* = 3) for each formulation, and the results are presented as mean values with standard deviation (error bars) in Figure 3.

### 2.5. Composting-Oriented Screening

End-of-life behavior was evaluated through an aerobic composting experiment designed to approximate practical biodegradation conditions. Compost “recipes” were developed to achieve an overall C/N ratio close to 30:1 by combining food waste (vegetable peels) [44,45], fresh grass clippings, soil, and fragments of the investigated biocomposites; multiple composting units were prepared in parallel, including containers with larger surface area (trays) to facilitate mixing/aeration and pots representing a more static configuration. The determination of the carbon-to-nitrogen (C/N) ratio in the sludge incorporated into the biocomposites was based on literature data regarding the C/N ratios of wood fibers, fresh grass, vegetable peels, and soil, as well as on chemical analyses of SCG and ALS. On this basis, the calculated C/N ratio of the investigated biocomposites ranged from 290 to 360. Using these values, together with the moisture content of each waste fraction, the mass proportions of the individual components in the three mixtures were determined to achieve an overall C/N ratio of 30:1 for the entire composting mixture (Table 1).

**Figure 2:**
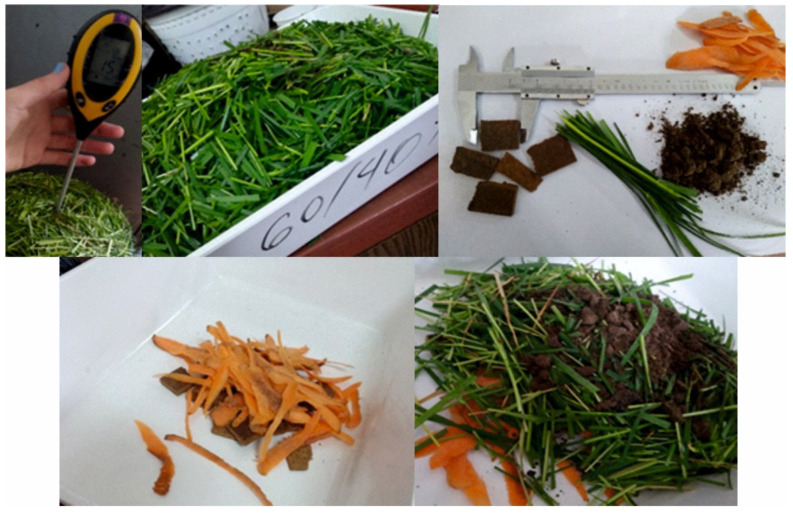
Сomposting setup (pots vs. trays), layering concept, and monitoring approach.

The compost mixtures were assembled in layers (biocomposite fragments placed in the lower layers, followed by alternating layers of food waste, soil, and grass), moistened, and then maintained over the test period by periodic watering and, for the tray units, intermittent mixing to enhance oxygen supply. Temperature was monitored during the composting period as an indicator of biological activity and process progression (Figure 2).

Unless stated otherwise, results are reported as mean values. For colorimetric measurements, three repeated readings were performed per formulation (*n* = 3) and are reported as mean ± standard deviation. The composting assessment is presented as an exploratory screening based on temperature monitoring and visual observations.

## 3. Results and Discussion

### 3.1. Color Development and Aesthetic Implications in SCG–ALS Wood-Fiber Biocomposites

**Figure 3:**
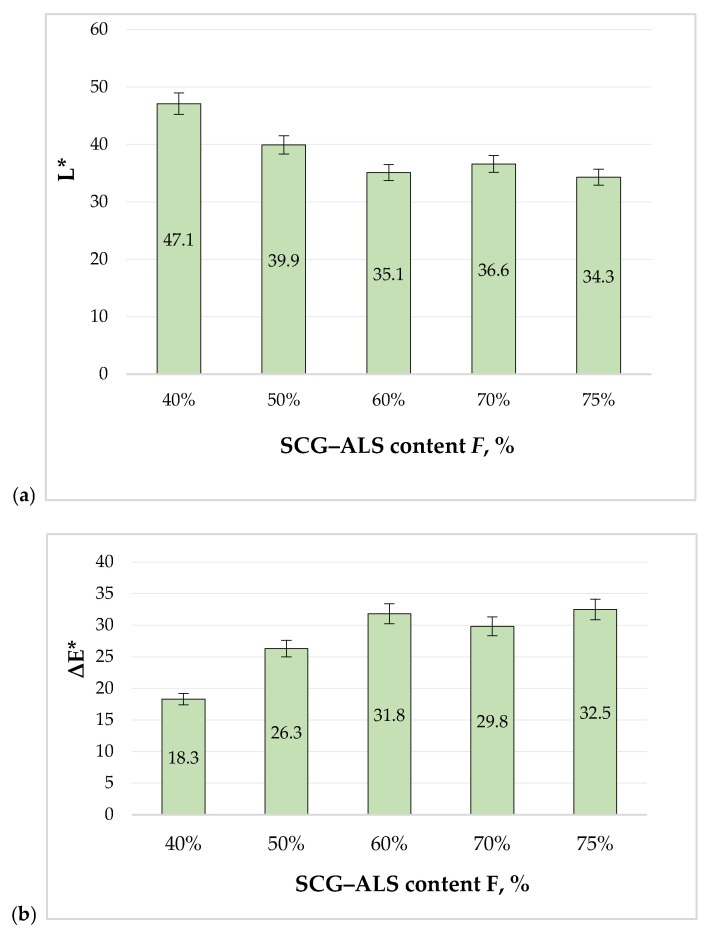
Formulation-dependent CIELAB parameters of SCG–ALS panels as a function of total SCG–ALS content (*F*, %): (**a**) lightness L* and (**b**) overall color difference ΔE*ab relative to the reference (standard). Bars show mean values; error bars indicate the standard deviation of three repeated measurements (*n* = 3).

The incorporation of spent coffee grounds (SCGs) and ammonium lignosulfonate (ALS) produced a strong and systematic shift in appearance relative to the reference material. Across the formulation range with total SCG + ALS contents of 40–75 wt.% (oven-dry basis), lightness (L*) decreased markedly, confirming progressive darkening as the biobinder fraction increased. The measured L* values ranged from 47.1 at 40 wt.% total SCG + ALS to 34.3 at 75 wt.% total SCG + ALS, while the total color difference (ΔE*ab*) *increased from 18.3 to 32.5. These* Δ*E*ab values correspond to large visual differences, indicating that SCG–ALS biocomposites develop a distinctly darker “coffee-toned” visual identity compared with the reference (Figure 3).

This trend also suggests a tendency toward appearance stabilization at the highest loadings. In particular, the two darkest variants exhibit very similar CIELAB coordinates (P70: L* = 35.1, a* = 5.0, b* = 10.7; P75: L* = 34.3, a* = 5.4, b* = 10.7), corresponding to ΔE*ab ≈ 0.89 between them, i.e., below a just-noticeable difference under controlled viewing. Therefore, beyond ~70 wt.% total SCG + ALS, further increases in the SCG–ALS fraction are expected to have only marginal influence on perceived surface color, while formulation choices can be guided primarily by mechanical performance targets and end-of-life considerations.

Beyond the magnitude of darkening, the chromatic coordinates also shifted with increasing SCG + ALS content. The reduction in a* and b* at higher binder fractions indicates a move toward a deeper brown/black tone, consistent with the intrinsic pigmentation of SCG and possible thermally induced transformations during hot pressing. Comparable trends are widely reported in SCG-filled polymer composites, where increased SCG loading generally decreases lightness and yields brown-to-dark coloration. In polypropylene (PP) biocomposites, SCG serves as a natural filler and colorant; although treatments and compatibilizers improve interfacial adhesion and mechanical performance, the characteristic dark appearance persists as a persistent feature of SCG incorporation [24]. Similar observations hold for PLA-based systems, where the presence of SCG affects both property profiles and the composite’s visual appearance, often necessitating design choices that accept or leverage darker shades [25]. Recent work on SCG–PLA composites for eco-oriented products and building-related elements also describes SCG’s natural pigmentation as an inherent material attribute that influences product aesthetics and may reduce the need for artificial pigments, particularly in applications where a “natural” appearance is valued [26,27]. In studies targeting FDM additive manufacturing, SCG-containing PLA composites are frequently presented as sustainable, visually distinctive materials, while noting that higher SCG content can intensify darkening and may require coatings or post-processing when lighter visual outcomes are desired [28].

For wood-fiber biocomposites, the aesthetic effect can be even more pronounced than in many polymer matrices because the baseline fiber substrate is relatively light. This amplifies contrast and makes the formulation-dependent color shift highly visible even at moderate SCG + ALS levels. In practical terms, the strong and predictable influence of SCG + ALS content on L* and ΔE* can be interpreted as a “design lever” that enables tailoring of appearance through formulation control. At the same time, the results emphasize that light-colored product targets would likely require either lower SCG + ALS contents, surface finishing, or a layered product concept in which SCG-rich material is used as a core with a lighter surface layer. These considerations are aligned with broader trends in lignin- and residue-based materials research, where aesthetics and perception increasingly influence acceptance alongside mechanical and environmental performance [8,9].

Raw-material variability is expected to be one of the main sources of scatter in the measured CIELAB coordinates, particularly the lightness (L*) of SCG-containing panels. In the case of spent coffee grounds, differences in roasting degree (light–dark), coffee origin/blend, grinding size distribution, brewing/extraction history, and residual extractives and lipids can modify both the intrinsic chromophores and the optical scattering behavior of the composite surface. Similarly, lignosulfonate-based binders may exhibit batch-to-batch variation related to feedstock (wood species), pulping/sulfite conditions, solids content, pH, ash/mineral fraction, and molecular-weight distribution, which can affect the binder color, curing kinetics, and ultimately the final surface appearance. Therefore, the colorimetric response observed here should be interpreted within the context of the specific material batches and processing conditions used.

From an implementation perspective, our formulation–color relationship can be viewed as an empirical calibration within a defined “process window” (raw-material characteristics, target moisture, pressing schedule, and surface preparation). For industrial scale-up, maintaining a constant L* target would benefit from routine incoming-batch screening (e.g., moisture, particle-size distribution, and simple colorimetric checks of SCG and biobinder) coupled with rapid verification on short-pressed coupons or early production boards. Such measurements could be used in a closed-loop manner to update the model coefficients for each batch and to apply small composition corrections (e.g., adjusting SCG fraction and/or biobinder dosage within predetermined limits) to compensate for raw-material drift while preserving both aesthetics and performance. Importantly, this approach emphasizes that the model is predictive and actionable under controlled conditions, but recalibration is recommended whenever raw-material sources or key process parameters change.

In terms of visual perception, the color differences of the SCG–ALS panels relative to the light reference are substantial. As a practical guideline, ΔE*ab* values around ~1 correspond to a just-noticeable difference under controlled viewing, while differences above ~2–3 are typically noticeable to an average observer, and values above ~5 are generally considered clearly visible in consumer products. In our case, the measured Δ*E*ab values ranged from 18.3 to 32.5, driven mainly by a pronounced decrease in lightness (ΔL* = −18.3 to −31.1), confirming a distinctly darker “coffee-toned” appearance. Within the SCG-rich formulations, however, the color coordinates tend to converge: the two darkest variants in our dataset show very close CIELAB values (ΔE*ab < 1 between them), suggesting that beyond a certain SCG–ALS level, the surface color becomes comparatively stable rather than continuously shifting. Therefore, the “golden mean” between high biobinder content and acceptable aesthetics is application-dependent: for light-color targets, reduced SCG–ALS or layered/finished concepts are needed, whereas for products that intentionally adopt a dark, natural look, higher biobinder contents can be selected primarily based on performance and end-of-life considerations without substantially changing the perceived color.

### 3.2. Composting Response Under Mesophilic Laboratory Conditions and Comparison with SCG Composting Literature

The composting experiment was designed as an exploratory, composting-oriented screening that reflects a realistic limitation of small-scale composting: reduced heat retention and correspondingly lower peak temperatures. Over 30 days, the temperature in the compost units remained predominantly within the mesophilic range, closely tracking ambient conditions and without a sustained thermophilic phase. Such temperature profiles are consistent with the expected behavior of small compost masses and shallow container geometries, where heat losses prevent the self-heating needed to reach >50 °C, even when microbial activity is present [20]. Differences between container types were modest; tray units occasionally showed slightly higher peaks than pot units, plausibly due to easier mixing and improved aeration, which can enhance aerobic decomposition dynamics. This is consistent with the expected effect of improved aeration/mixing in trays, which can locally intensify aerobic activity even in small composting units. It should be emphasized that the present setup is not intended to provide a standardized compostability classification. Because the composting masses were small and operated under laboratory conditions without forced aeration or controlled turning, the process remained predominantly mesophilic and cannot be directly compared to industrial composting regimes. Therefore, the outcomes are interpreted as qualitative evidence of biological susceptibility under moist, nutrient-rich conditions rather than as quantified biodegradation/disintegration rates. This framing defines the next step as standardized testing with quantitative endpoints (e.g., disintegration metrics, mass loss and/or CO_2_ evolution proxies, and compost maturity/toxicity indicators).

Despite the absence of thermophilic temperatures, visible microbial colonization and progressive surface changes in embedded fragments were observed over the test period, indicating the biocomposites’ susceptibility to biological activity under moist, nutrient-rich conditions. This is consistent with the understanding that biodegradation in composting can proceed under mesophilic conditions, although typically at slower rates than in optimized industrial composting systems. Importantly, studies explicitly focused on SCG co-composting show that composting outcomes depend strongly on recipe composition and process management. Santos et al. evaluated SCG incorporation at increasing rates (including high SCG fractions). They reported that SCG can be successfully composted while monitoring gaseous emissions and compost quality, with outcomes sensitive to mixture composition and composting progression [21]. Liu and Price compared in-vessel composting, aerated static pile composting, and vermicomposting for SCG management and highlighted that system design governs stabilization timelines and nutrient conservation, with faster throughput and tighter process control typically achieved in in-vessel systems [20]. These comparative findings help contextualize the present results: the laboratory container approach used here is closer to a low-intensity, home-compost analog than to industrial composting, and therefore it should be expected to display slower kinetics and lower peak temperatures.

Additional insight comes from studies comparing the effects of composting and vermicomposting on microbial activity and substrate transformation, demonstrating that different biological pathways can yield distinct stability and maturity outcomes [19]. From a circularity standpoint, this is particularly relevant because SCG and other organic residues can contain compounds associated with phytotoxicity if applied to soil without sufficient stabilization; composting is often recommended as a mitigation route to improve safety and agronomic suitability [21]. Soil-amendment studies further indicate that composting time influences early plant development and soil interaction, highlighting the practical need to ensure adequate stabilization before circular return to soil [18]. In agronomic contexts, SCG application can improve certain soil and plant nutrient parameters, but responses depend on dosage, soil type, and treatment level, reinforcing that “SCG-derived” materials should be assessed with attention to processing and maturity rather than assumed benign by default [17,18].

In the current system, the biocomposites include ALS, a water-soluble sulfonated lignin derivative that is chemically distinct from untreated lignin. In adhesive research, ALS is recognized as a promising bio-based component but often requires formulation engineering to achieve the desired performance in wood composites [5]. Under composting conditions, the hydrophilic functionality and fine particulate structure contributed by SCG and ALS may promote water uptake and microbial accessibility in the near-surface region, which is consistent with the observed surface colonization in moist compost. Nevertheless, because the present composting assessment was intentionally exploratory and qualitative, the results should be interpreted as evidence of biological susceptibility rather than as a standardized compostability classification.

**Figure 4:**
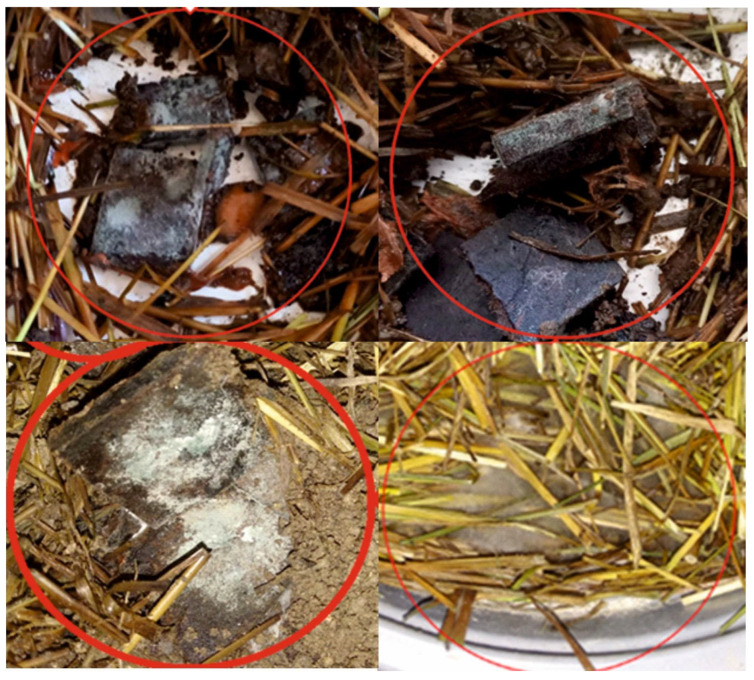
Colonization of compostable mixtures by filamentous fungi at different component ratios.

As a result of hot pressing at 160 °C for 15 min (three-stage pressure schedule: 1.8 MPa for 1 min, 0.6 MPa for 10 min, and 0.4 MPa for 4 min), the freshly manufactured panels were expected to exhibit a substantially reduced microbial load. However, recolonization can occur during storage/handling, and composting introduces a high microbial inoculum, high moisture, and readily available nutrients. When the SCG–ALS boards were incorporated into compost mixtures with fresh grass and carrots, soil, and other organic fractions, conditions favored the development of bacteria, actinomycetes, and filamentous fungi. Consistent with this, the photographic record shows colonization by filamentous fungi, with more pronounced growth in the 50/50 and 60/40 variants (wood fibers to SCG + ALS mixture), as shown in Figure 4. Coffee processing wastes, including spent coffee grounds and other coffee-derived residues, contain substantial amounts of organic compounds such as lipids, amino acids, polyphenols, polysaccharides, minerals, and others [44,45]. Under conditions of biological degradation and composting, coffee residues may be colonized by diverse microorganisms. Filamentous fungi belonging to the genera Aspergillus and Penicillium have demonstrated the ability to grow on coffee residues and to actively participate in the degradation of organic matter during composting [44].

The composting feedstocks provide complementary substrates that can support the functional succession typically reported for aerobic composting. Easily degradable plant residues (fresh grass and root crops) supply soluble carbohydrates and proteins that stimulate early-stage heterotrophic and fermentative microorganisms, while soil acts primarily as an inoculum and buffer, contributing diverse bacterial, actinomycete, and fungal communities and supporting key nitrogen-cycle transformations. As composting progresses, lignocellulosic materials increasingly act as both a bulking agent (improving porosity and aeration) and a longer-term carbon source; their decomposition is commonly associated with cellulolytic/hemicellulolytic activity followed by slower transformation of lignin-rich fractions during later maturation stages [45,46,47,48,49,50].

SGS contributes organic carbon and nitrogen together with phenolic/caffeine compounds that may modulate microbial activity: at high local concentrations, some components can inhibit sensitive groups, whereas in balanced mixtures, SCG can sustain active microbial communities and a noticeable fungal phase [50,51,52,53,54,55,56,57]. In the present composites, ammonium lignosulfonate (ALS)—a sulfonated, water-soluble lignin derivative—is expected to behave as a relatively slowly degradable aromatic component; its transformation in compost environments is generally attributed to ligninolytic fungi and actinomycetes producing oxidative enzymes such as laccases and peroxidases [58,59]. Overall, these literature-based considerations provide a mechanistic context for the observed surface colonization and degradation phenomena. At the same time, quantitative microbiological profiling would be required to confirm the community composition in the present composting setups.

In related composting variants based on plant residues and wood-derived fractions, culture-based assessments commonly report high counts of non-spore-forming bacteria and bacilli, with actinomycetes and filamentous fungi typically present at lower counts depending on process conditions and compost maturity [59,60,61,62,63,64,65,66,67,68].

The selected substrate combinations are therefore expected to support aerobic decomposition under the applied laboratory conditions. However, compost maturity and agronomic safety cannot be inferred from visual observations alone; future work should include maturity indicators (e.g., pH and C/N evolution, respiration/CO_2_-based activity metrics) and phytotoxicity screening (e.g., germination index) to verify whether the resulting compost is suitable for soil application and to better quantify the role of SCG–ALS biocomposite fragments in the process.

### 3.3. Implications for Circular Design and Positioning Among SCG Valorization Routes

When combined with the preceding performance-focused development work [4], the current findings add two application-facing layers: aesthetics and end-of-life plausibility. The strong color shift confirms that the SCG–ALS fraction largely defines the visual identity of the biocomposites, which can be beneficial when targeting naturally dark products but may require finishing strategies for light-toned applications. From an end-of-life perspective, the onset of visible degradation phenomena under composting-like conditions suggests that composting-oriented circularity routes may be feasible, at least for manufacturing residues and potentially for post-use waste, provided that downstream processing is appropriately controlled.

Within the broader SCG valorization literature, composting represents only one of several viable routes. SCG is also widely assessed for energy recovery and fuel products, including pelletization and carbonization to increase heating value [15,16], and for conversion processes such as hydrothermal liquefaction aimed at bioenergy and biochemical streams [14]. In addition, SCG is explored in higher-value functional materials such as activated adsorbents [13] and engineered carriers (e.g., cellulose-based supports) for biotechnological applications [12]. Reviews that synthesize these options emphasize that selecting the “best” pathway depends on local infrastructure, scale, and product goals [8,9]. Against this landscape, SCG–ALS wood-fiber biocomposites offer a materials-focused valorization route that can couple waste utilization with functional products, but robust circularity claims require further verification beyond qualitative observation.

In this landscape, SCG–ALS wood-fiber biocomposites represent a “materials route” that can lock residues into functional products with a defined visual identity and potential service life, rather than treating SCG solely as feedstock for energy recovery or direct biological processing. Accordingly, the most appropriate circular pathway should be selected case-by-case: long-lived interior products may prioritize durability and controlled aesthetics, whereas short-lived items and manufacturing residues may favor end-of-life options such as controlled biological treatment, provided that standardized verification confirms environmental compatibility.

Accordingly, the next step toward application-ready exploratory composting-oriented screening is standardized compostability testing that quantifies disintegration and biodegradation (e.g., mass loss, fragmentation metrics, CO_2_ evolution proxies, and maturity/toxicity screening of compost output). Such work would also clarify whether ALS-containing formulations influence compost quality in ways relevant to soil application, a question that aligns with the broader literature on SCG stabilization and soil compatibility [17,18,19,21]. At the current stage, the present findings should therefore be interpreted as exploratory screening results and not as evidence for compliance with any compostability standard.

Surface finishing is another practical factor that can influence the end-of-life behavior of SCG–ALS panels. The present composting-oriented screening was performed on uncoated fragments; therefore, the observed disintegration trends reflect direct contact between the biocomposite surface and the composting matrix. In real products, oils, varnishes, waxes, laminates, or polymeric coatings may partially seal the surface, reduce moisture uptake, and limit microbial access, which is expected to slow down disintegration and biodegradation under mesophilic composting conditions. Consequently, composting as an end-of-life option is most directly relevant to uncoated boards, production residues (cuts, sanding dust, off-spec panels), and short-lived applications where protective coatings are not required. A systematic assessment of common finishes—including bio-based and potentially compostable coatings—under standardized compostability protocols is identified as an important direction for future work.

## 4. Conclusions

This follow-up study complements prior performance-focused research on SCG–ALS wood-fiber biocomposites by addressing two critical dimensions: aesthetic predictability and end-of-life feasibility. Formulation-resolved colorimetry shows that the SCG-ALS fraction strongly governs visual identity, producing systematic darkening with increasing total SCG + ALS content (lower L* and larger ΔE* relative to the reference), thereby enabling aesthetics-aware formulation design and intentionally positioning “coffee-toned” biocomposite products with improved appearance reproducibility. A 30-day, small-scale composting screening performed under predominantly mesophilic laboratory conditions revealed visible microbial colonization and progressive surface degradation of embedded fragments. These observations indicate susceptibility to biological activity in moist, nutrient-rich environments and suggest composting-oriented end-of-life routes as a plausible direction at an exploratory level—potentially most relevant for production residues or short-lived applications.

The main limitations of the study are that appearance was quantified only through short-term surface color under controlled conditions, without accounting for raw-material variability, finishing/coatings, UV/thermal aging, or in-service soiling. Panels were manufactured at laboratory scale within a single processing window, limiting direct transferability to industrial variability. The composting assessment was qualitative and short-term and did not include standardized measures of biodegradation/disintegration, compost maturity, ecotoxicity, or mass/CO_2_ evolution; therefore, the material cannot be classified as compostable under any standard based on the present results.

Future work will focus on (i) decoupling the SCG/ALS ratio within the binder system beyond the present 50:50 approach to identify formulations that optimize both aesthetics and bonding performance; (ii) deeper thermal and thermomechanical characterization of the adhesive mixtures and panels (e.g., detailed TGA/DTG/DSC-based profiling and curing/softening behavior) to link composition with processing stability and durability; and (iii) moving from exploratory composting screening to standardized, quantitative compostability validation, including disintegration and biodegradation metrics (e.g., mass loss/fragmentation and CO_2_-evolution-based indicators) together with compost maturity and ecotoxicity endpoints. In parallel, the influence of realistic surface finishes/coatings—including bio-based and potentially compostable coatings—on end-of-life behavior will be assessed, since many commercial products require protective treatments that may hinder microbial access.

Overall, the key contribution is an integrated, formulation-dependent mapping of objective color metrics together with an initial end-of-life screening for the SCG–ALS wood-fiber system. Practically, the resulting color–formulation relationships can be used as design and quality-control targets for manufacturers aiming to deliver consistent, intentionally dark “coffee-toned” panels, while composting observations help frame realistic end-of-life scenarios for residues and short-lived products. Looking forward, future work should scale the process window and raw-material variability, evaluate appearance stability under aging and finishing, and conduct standardized compostability/biodegradation testing, including maturity and ecotoxicity endpoints, to substantiate circularity claims and support application-specific certification and product development.

## 5. Patents

Savov, V.P.; Yusein, J.; Kostadinova-Slaveva, A.G.; Brankova, S.R. Eco-friendly biocomposite material. Bulgarian Utility Model No. 4490, 19 June 2023. (Original title in Bulgarian: “Екoлoгичен биoкoмпoзитен материал”.) The utility model protects an application concept; the present article reports independent experimental results and statistical analyses not disclosed in the registration document.

## Figures and Tables

**Table 1:** Composition of different waste fractions depending on the biocomposite formulation (dry-matter basis, DM, %).

Waste Types Used in the Composting Process	Sample 1	Sample 2	Sample 3
		DM, %	
Fruit and vegetable peels	19.2	6.7	12.8
Grass	57.7	66.7	56.4
Biocomposites	3.8	4.4	5.1
Soil	19.2	22.2	25.6

**Note:** The values in Table 1 represent the nominal composition of the composting mixtures on a dry-matter basis, calculated from weighed input materials and their determined dry matter (DM). As these are recipe-derived (deterministic) proportions rather than repeated outcome measurements, a standard deviation is not applicable.

## Data Availability

The original contributions presented in this study are included in the article. Further inquiries can be directed to the corresponding authors.
